# Taming conformational entropy for low-cost and high-performance organic photovoltaics

**DOI:** 10.1093/nsr/nwag355

**Published:** 2026-06-11

**Authors:** Yongsheng Chen

**Affiliations:** State Key Laboratory and Institute of Elemento-Organic Chemistry, The Centre of Nanoscale Science and Technology and Key Laboratory of Functional Polymer Materials, Renewable Energy Conversion and Storage Center (RECAST), Frontiers Science Center for New Organic Matter, College of Chemistry, Nankai University, China

**Figure 1.**
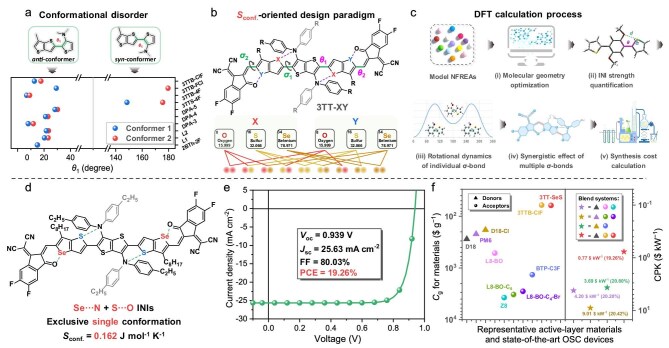
(a) Conformational disorder posed by rotatable σ-bonds. (b) Design strategy for model nonfused-ring electron acceptors (NFREAs). (c) Flowchart of DFT calculation procedures. (d) Chemical structure, single-crystal conformation, and calculated *S*_conf._ of 3TT-SeS. (e) Photovoltaic performance of the champion 3TT-SeS-based device. (f) Statistics of power generation cost for active-layer materials. Adapted with permission from Ref. [4].

The pursuit of ubiquitous solar energy via lightweight, flexible organic photovoltaics (OPVs) has long been constrained by a fundamental dilemma: the trade-off between material cost and device performance [1]. While fused-ring electron acceptors (FREAs) deliver high power conversion efficiencies (PCEs) beyond 20%, their complex synthesis hinders large-scale commercialization. In contrast, nonfused-ring electron acceptors (NFREAs) provide a low-cost alternative featuring simple-structured backbones and concise synthetic routes [2]; however, their inherent structural flexibility leads to conformational disorder (Fig. 1a), which in turn increases static disorder in the solid state, thereby compromising charge transport and ultimately limiting the photovoltaic performance. Instead of solely focusing on backbone planarization [3], addressing this intrinsic disorder holds the key to unlocking the full potential of NFREAs.

In a transformative study inspired by protein folding, Huang and co-workers introduce an innovative design paradigm: conformational entropy (*S*_conf._) tuning [4]. They posit that suppressing *S*_conf._ in NFREAs is as crucial for achieving ordered molecular packing and efficient charge transport as it is for polypeptide folding into biologically functional proteins [5]. This represents a conceptual leap beyond prior approaches, targeting the thermodynamic origin of structural disorder in organic semiconductors.

To realize this vision, the team employed a computer-aided molecular design method (Fig. 1b and c). By strategically combining varied intramolecular noncovalent interactions (INIs), they identified an optimal NFREA named 3TT-SeS featuring balanced interactions (Se$\cdots$N and S$\cdots$O INIs; Fig. 1d). This molecule achieves an ultralow *S*_conf._, and adopts a single, exclusive planar conformation. This ‘conformer-free’ state translates into a highly ordered self-assembled structure in the solid state, as confirmed by single-crystal analysis and morphology characterization. Consequently, photovoltaic devices based on 3TT-SeS achieved a record-breaking PCE of 19.26% (certified 18.75%) for NFREA-based systems (Fig. 1e), bridging the performance gap with costly FREAs. More impressively, the system demonstrates superior economic viability, with a calculated power generation cost as low as $0.77 per kW, dramatically undercutting the state-of-the-art FREA-based counterpart (Fig. 1f).

The significance of this work stretches well beyond a single material or efficiency record: it establishes *S*_conf._ as a quantitative, predictive descriptor to guide the molecular design of organic semiconductors. By translating this fundamental thermodynamic parameter into practical materials science applications, the authors deliver a universal blueprint for resolving the longstanding cost-performance paradox in OPVs. This low-*S*_conf._ design framework represents a paradigm shift—from empirical structural optimization to thermodynamics-guided molecular engineering—thus opening a clear, scalable and industrially viable pathway for advancing high-performance organic electronic materials [6].
